# Emerging Thrombolysis Technologies in Vascular Thrombosis

**DOI:** 10.3390/jcm14217758

**Published:** 2025-11-01

**Authors:** Bingwen Eugene Fan, Yixin Jamie Kok, Chuen Wen Tan, Yu Yue Hew, Brandon Jin An Ong, Benjamin Yong-Qiang Tan, Winnie Z. Y. Teo, Rinkoo Dalan, Yen Lin Chee, Eng Soo Yap

**Affiliations:** 1 Department of Haematology, Tan Tock Seng Hospital, 11 Jalan Tan Tock Seng, Singapore 308433, Singapore; 2 Department of Laboratory Medicine, Khoo Teck Puat Hospital, Singapore 768828, Singapore; 3 Lee Kong Chian School of Medicine, Nanyang Technological University, Singapore 308232, Singapore; 4 Yong Loo Lin School of Medicine, National University of Singapore, NUHS Tower Block 1E Kent Ridge Road, Singapore 119228, Singapore; 5 Department of General Medicine, Tan Tock Seng Hospital, 11 Jalan Tan Tock Seng, Singapore 308433, Singapore; 6 Department of Haematology, Singapore General Hospital, Singapore 169608, Singapore; 7 Department of Neurology, National University Hospital, Singapore 119228, Singapore; 8 Fast and Chronic Program, Alexandra Hospital, Singapore 159964, Singapore; 9 Department of Haematology-Oncology, National University Cancer Institute, Singapore 119074, Singapore; 10 Department of Endocrinology, Tan Tock Seng Hospital, 11 Jalan Tan Tock Seng, Singapore 308433, Singapore; 11 Department of Laboratory Medicine, National University Hospital, Singapore 119228, Singapore; 12 Department of Laboratory Medicine, Ng Teng Fong General Hospital, Singapore 609606, Singapore

**Keywords:** electromechanical, magnetic, microbots, nanoparticles, phototherapy, thrombolysis, thrombosis, ultrasound

## Abstract

**Background/Objectives**: Thrombotic diseases, such as ischemic stroke, acute myocardial infarction, and venous thromboembolism, are leading causes of global morbidity and mortality. Traditional thrombolytic therapies like systemic tissue plasminogen activator (tPA) are limited by bleeding risks, poor targeting, and inconsistent efficacy. This review explores emerging non-pharmacological technologies aimed at overcoming these challenges through targeted, minimally invasive thrombolysis. **Methods**: A narrative synthesis of recent advancements was conducted, focusing on six innovative approaches: ultrasound-mediated thrombolysis (UMT), microrobots, electrothrombectomy, photothrombectomy, magnetic targeted thrombolysis, and nanotechnology. Preclinical and clinical studies were reviewed to assess mechanisms, efficacy, safety, and translational potential, prioritizing technologies with demonstrated success in animal or early human trials. **Results**: Technologies like microbubble-enhanced UMT, magnetically actuated microrobots, and fibrin-targeted nanoparticles showed promising results. UMT improved recanalization in ischemic stroke and pulmonary embolism, while electrothrombectomy demonstrated safe, effective clot extraction in human trials. However, challenges remain in scalability, biocompatibility, and clinical integration, with microrobots and photothrombectomy still in preclinical stages. **Conclusions**: Emerging thrombolysis technologies offer safer, more targeted alternatives to conventional treatments. Clinical adoption will depend on overcoming translational hurdles, including large-scale trials, miniaturization, and interdisciplinary collaboration, with a focus on hybrid approaches and real-time imaging integration.

## 1. Introduction

Thrombotic diseases are a leading cause of morbidity and mortality worldwide, accounting for 1 in 4 deaths worldwide in 2010 and are the leading cause of mortality, significantly impacting global health systems [1]. Annually, approximately 7.3 million people experience strokes globally, with acute ischemic strokes (AIS) accounting for 65.3% of cases [2]. A subset of AIS cases arises from inherited or acquired thrombophilic states, including Factor V Leiden mutation, prothrombin G20210A, and antiphospholipid syndrome. Acute myocardial infarction (AMI) approaches 3 million people worldwide, with more than one million deaths in the United States annually, constituting a major cardiovascular burden [3]. In addition, AIS is a leading cause of long-term disability, with nearly 40% of survivors experiencing functional dependence at 6 months, while AMI survivors have a 25% risk of recurrent cardiac events within five years. Peripheral artery disease (PAD) affects over 200 million people globally, often leading to ischemia and limb loss [4]. Deep vein thrombosis (DVT) and pulmonary embolism (PE), collectively known as venous thromboembolism (VTE), contribute to 10 million cases annually, with PE carrying a mortality rate exceeding 30% if untreated [5]. Additionally, complications in dialysis patients, such as arteriovenous (AV) fistula thrombosis, exacerbate the healthcare burden and disrupt essential treatment [6]. Traditional thrombolytic therapies, including systemic administration of tissue plasminogen activator (tPA) and anticoagulants, remain the cornerstone for managing these conditions but are fraught with limitations. High bleeding risks, such as intracranial haemorrhage, occur in 4.9% of patients undergoing thrombolytic therapy for ischemic stroke [7]. Systemic side effects and the inability to precisely target thrombi further undermine treatment efficacy and safety, particularly in diverse vascular territories. These limitations necessitate a paradigm shift toward innovative thrombolysis techniques that minimize bleeding complications and enhance targeting precision.

**Figure 1:**
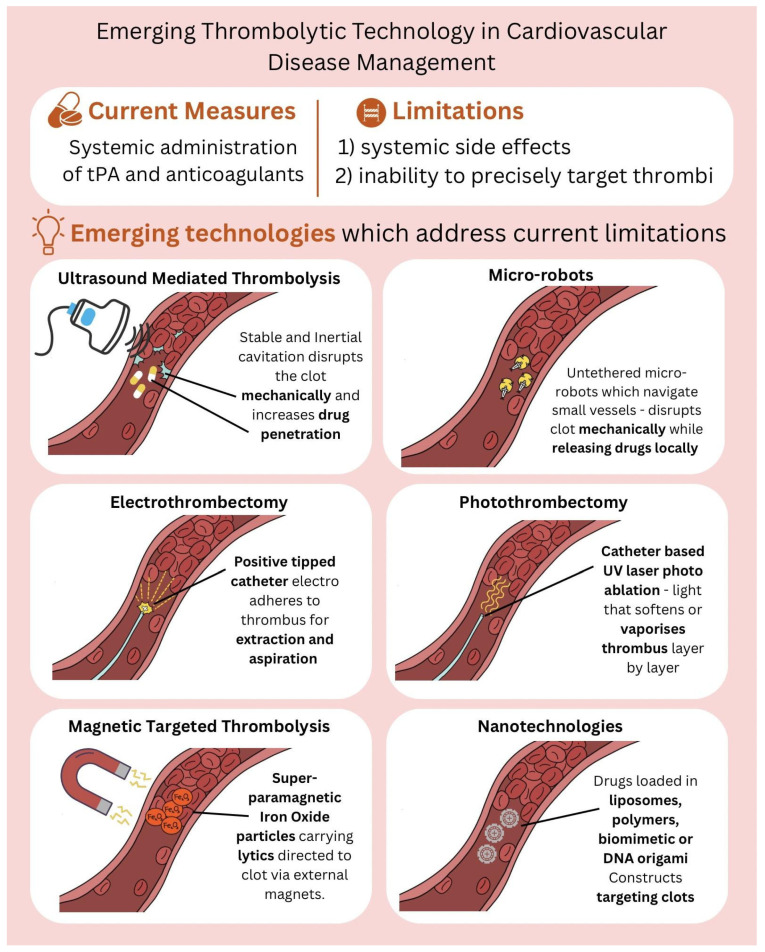
Schematic representation of emerging non-pharmacological thrombolysis strategies. Sonothrombolysis uses ultrasound-induced cavitation to enhance clot dissolution. Magnetic-directed thrombolysis employs iron oxide nanoparticles guided by external magnetic fields. Photo- and electrothrombectomy involve light-based or electrical energy to ablate or extract thrombi. Microrobots navigate vasculature to mechanically disrupt clots, while nanoparticles enable targeted delivery and controlled release of thrombolytic agents. Abbreviation: tPA, tissue plasminogen activator.

This review is the first to synthesize six emerging non-pharmacological and hybrid thrombolytic platforms, namely ultrasound-mediated thrombolysis, microrobots, electrothrombectomy, photothrombectomy, magnetic targeted thrombolysis and nanotechnology-based solutions (Figure 1), across both arterial and venous thromboses, comparing mechanisms, translational maturity, and regulatory readiness levels. It provides an integrated framework linking device engineering to clinical application, which has not been previously consolidated in a single review. By discussing these advances, we aim to provide insights into the next generation of thrombolysis interventions, bridging gaps in traditional management approaches.

## 2. Methods

To comprehensively evaluate the current landscape of emerging thrombolysis technologies in cardiovascular disease management, we conducted a scoping review across three major biomedical databases: PubMed, Embase, and Scopus. The search focused on identifying preclinical and clinical studies, reviews, and technological reports published in English from January 2000 to May 2025. Key search terms included combinations of “thrombolysis”, “ultrasound-mediated”, “sonothrombolysis”, “microrobots”, “magnetically guided thrombolysis”, “electrothrombectomy”, “photothrombectomy”, “nanotechnology,” “theranostics,” and “targeted thrombolytic delivery.” Boolean operators (AND, OR) were employed to expand or narrow results, and Medical Subject Headings (MeSH) terms were incorporated where applicable. Priority was given to studies that evaluated novel mechanisms of clot dissolution, device-based or non-pharmacological interventions, targeted delivery platforms, and early-phase human trials. References from retrieved articles were screened manually to identify additional relevant publications. The synthesis of findings was guided by their translational relevance, mechanistic innovation, safety profile, and potential for integration into current thrombolytic protocols.

## 3. Thrombolysis Technologies

### 3.1. Ultrasound-Mediated Thrombolysis (Sonothrombolysis): Mechanism, Technologies, and Clinical Applications

Ultrasound-mediated thrombolysis (UMT), also known as sonothrombolysis, represents a significant advancement in the management of thrombotic diseases (Table 1 and Table 2). It uses therapeutic ultrasound to achieve thrombus dissolution by leveraging mechanical and acoustic effects such as cavitation, acoustic radiation force, and microstreaming [8]. These effects enhance clot fragmentation and drug delivery, making UMT a promising approach in both invasive and non-invasive contexts while mitigating the limitations of conventional thrombolytic approaches. UMT is increasingly recognized for its versatility across various clinical contexts, including AIS [9], AMI [10], venous thromboembolism (VTE) [11,12,13], and arteriovenous fistula (AVF) thrombosis [14].

UMT operates through multiple mechanisms that facilitate clot lysis. Cavitation is perhaps the most studied and involves the formation, oscillation, and collapse of microbubbles within a liquid medium due to the alternating high-pressure and low-pressure cycles of ultrasound waves [15,16]. Cavitation plays a dual role in UMT: enhancing the mechanical disruption of thrombi and facilitating the penetration of thrombolytic agents. It is typically categorized into two types: stable cavitation and inertial (or transient) cavitation, both of which contribute distinct effects in clot lysis [17,18]. Stable cavitation occurs when microbubbles oscillate in size without collapsing under the influence of ultrasound waves. These oscillations generate localized fluid motion, termed microstreaming, which exerts shear forces on the clot’s fibrin network. This loosens the clot structure, making it more susceptible to enzymatic or mechanical disruption. Stable cavitation also enhances the diffusion of thrombolytic agents, such as tissue plasminogen activator (tPA), by creating microcurrents that drive the drugs deeper into the thrombus. Stable cavitation is commonly observed at lower ultrasound intensities and is often augmented by the introduction of ultrasound contrast agents like microbubbles [19] or nanodroplets [20]. These agents act as nuclei for cavitation, reducing the energy required to initiate oscillations. Inertial cavitation occurs when microbubbles grow rapidly during the low-pressure phase of ultrasound waves and violently collapse during the high-pressure phase [21]. This collapse generates localized high-energy events, including shock waves (High-pressure waves that mechanically fragment the thrombus), microjets (high-velocity liquid jets that penetrate and erode the clot structure) and localized heating (transient increases in temperature that may further weaken the clot matrix). Inertial cavitation typically requires higher ultrasound intensities and is more destructive than stable cavitation. While it can effectively fragment clots, excessive inertial cavitation poses risks of vascular injury [22], including endothelial damage and haemorrhage.

Recent advances in UMT technologies have addressed key limitations of conventional methods. Acoustic radiation force displaces thrombus material, enhancing thrombolytic drug infiltration. Microstreaming facilitates drug transport into the clot, while thermal effects can subtly augment enzymatic action. These processes are further enhanced by adjunctive technologies such as microbubbles, nanodroplets, and advanced contrast agents. Targeted microbubbles coated with ligands specific to thrombus-associated markers, such as glycoprotein IIb/IIIa, enhance site-specific thrombolysis, minimizing off-target effects [23]. Drug-loaded microbubbles provide localized thrombolytic agent delivery, reducing systemic exposure and associated risks of haemorrhage [24]. Phase-change nanodroplets, which vaporize under ultrasound energy, allow for improved clot penetration and reduced risk of vascular injury [25]. High-intensity focused ultrasound (HIFU) has emerged as a powerful tool, leveraging acoustic radiation force to achieve lysis without pharmacological agents [26]. Histotripsy, a non-thermal ablation method, induces cavitation to fractionate clots into fine debris, reducing risks of pulmonary embolism [27]. A refined version, microtripsy, further minimizes vascular damage by focusing energy more precisely [28].

The clinical applications of UMT are diverse and promising. In AIS, UMT has been shown to enhance recanalization rates, particularly when combined with microbubbles and tissue plasminogen activator (tPA). The CLOTBUST trial demonstrated improved outcomes in stroke patients treated with transcranial Doppler-mediated sonothrombolysis, without increasing bleeding risk [29]. In AMI, UMT shows potential for targeting coronary thrombi without necessitating high doses of systemic thrombolytics. Slikkerveer et al. conducted a pilot safety and feasibility study of low-dose tPA combined with US and microbubbles before percutaneous coronary intervention in patients with AMI. At angiography, 3 of 5 patients in the intervention group had Thrombolysis in Myocardial Infarction (TIMI) flow III versus 1 of 5 in the control group (*p* = 0.23) [30]. Xie et al. demonstrated that platelet targeted microbubbles combined with impulses from diagnostic ultrasound transducer improved epicardial recanalization rates and microvascular recovery in pigs with acute left anterior descending thrombotic occlusions [23]. For DVT and PE, catheter-based systems like EkoSonic Endovascular System (EKOS) have integrated forward-viewing transducers, achieving superior clot resolution with minimal complications and thus reducing clot burden and treatment duration [31]. Moreover, UMT’s application in AVF thrombosis holds promise for non-invasive restoration of patency, reducing the need for repeated surgical interventions [32].

Despite these advancements, challenges remain. Optimizing ultrasound parameters, such as acoustic intensity and duty cycle, as well as the selection design, and preparation of acoustic-responsive carriers have a decisive impact on the safety and therapeutic efficacy of acoustic thrombolysis [33]. The risk of vascular damage, haemorrhage, and embolization of clot debris underscores the need for meticulous preclinical and clinical studies. Device accessibility and cost also pose barriers to widespread adoption. Nonetheless, the integration of technologies such as dual-frequency HIFU and magnetic microbubbles offers potential solutions, further enhancing the safety and precision of sonothrombolysis. In essence, UMT represents a transformative approach to thrombolytic therapy, combining precision, safety, and efficacy. Innovations such as phase-change nanodroplets, histotripsy, and improved transducer designs hold the potential to redefine the standard of care in thrombotic disease management. Optimizing treatment parameters and ensuring safety will be pivotal to the clinical adoption of these promising technologies.

**Table 1:** Key Preclinical and Translational Studies on Ultrasound-Mediated Thrombolysis.

No.	Study/Reference	Model/Setting	Intervention & Mechanism	Key Findings	Translational Relevance
1	**Wildberger et al., *Cardiovasc Intervent Radiol*, 2001 [14]**	In vitro haemodialysis access model	Thrombus ablation in occluded haemodialysis access shunts utilizing ultrasound	Demonstrated effective clot fragmentation and removal without damage to vascular graft materials.	Provided early evidence supporting ultrasound as a safe, non-invasive tool for thrombus removal in vascular access.
2	**Datta S. et al., *Ultrasound Med Biol*, 2006 [15]**	In-vitro fibrin clot model	Diagnostic US (120 kHz–1 MHz) + tPA; cavitation monitoring	Cavitation dose correlated strongly with lysis rate enhancement	Defined quantitative cavitation–lysis relationship foundational for later energy-dosing strategies
3	**Maxwell et al., *Ultrasound Med Biol*, 2009 [27]**	In vivo canine clot model	Histotripsy using focused ultrasound pulses to induce controlled cavitation for mechanical clot fractionation.	Achieved complete thrombus fractionation without thermal injury or embolization; no vessel damage observed.	Established histotripsy as a non-thermal, non-pharmacologic thrombolytic modality.
4	**Chuang YH et al., *Ultrason Imaging*, 2010 [16]**	In-vitro fibrin clot dissolution	Controlled inertial cavitation via 1 MHz pulsed US	Enhanced fibrinolysis up to 3× baseline; optimal duty cycle identified	Clarified inertial cavitation threshold for enzymatic thrombolysis optimization
5	**Hua et al., *J Thromb Thrombolysis*, 2014 [24]**	Rabbit femoral artery thrombosis model	tPA-loaded targeted microbubbles activated with diagnostic ultrasound	Enhanced thrombus lysis and recanalization with reduced tPA dose compared to systemic therapy.	Demonstrated synergistic benefit of tPA-microbubbles with ultrasound for targeted thrombolysis.
6	**Zhang et al., *IEEE Trans Ultrason Ferroelectr Freq Control*, 2015 [28]**	In vitro thrombus model	Microtripsy technique using short, high-pressure ultrasound pulses	Enabled localized thrombus disintegration with minimized collateral damage and high reproducibility.	Improved control and safety profile over conventional histotripsy for clinical translation.
7	**Petit B et al., *Ultrasound Med Biol*, 2015 [17]**	In-vitro human clot model	Quantified stable vs. inertial cavitation using contrast microbubbles	Both cavitation types synergistically improved clot lysis; stable cavitation dominant	Established mechanistic framework distinguishing cavitation regimes
8	**Porter TR et al., *Invest Radiol*, 2017 [19]**	Porcine carotid thromboembolism	Diagnostic US-induced microbubble cavitation (no tPA)	Achieved >70% recanalization; no major hemorrhage	Proof of mechanical-only sonothrombolysis feasibility
9	**Suo D et al., *Ultrason Sonochem*, 2018 [18]**	Computational model	Multi-frequency cavitation modeling	Lower inertial threshold achieved with dual frequencies	Supports development of multi-frequency sonothrombolysis devices
10	**Hu B et al., *Int J Cardiol*, 2018 [20]**	Rat coronary microcirculation model	Acoustic phase-change dodecafluoropentane nanoparticles (PCNDs) + US	Significantly improved microvascular flow; no endothelial damage	Demonstrated translational feasibility of nanoparticle-assisted UMT
11	**Guo S et al., *Ultrason Sonochem*, 2019 [26]**	Rabbit femoral	Phase-change nanodroplet-assisted UMT	Reduced clot debris size; faster lysis kinetics	Advanced hybrid acoustic–nanocarrier approach
12	**Kim et al., *Ultrasound Med Biol*, 2020 [25]**	In vitro aged bovine clot models	Comparison of phase-change nanodroplets versus microbubbles under low-intensity ultrasound.	Phase-change nanodroplets achieved superior lysis efficiency in aged clots	Supports development of next-generation nanodroplet agents for resistant thrombi.
13	**Xie Y et al., *Ultrason Sonochem*, 2022 [22]**	Computational & in-vitro focused US field	Cavitation bubble–endothelium interaction	Predicted thresholds for vascular injury	Quantified safety envelope for high-intensity UMT
14	**Wang et al., *Front Bioeng Biotechnol*, 2022 [11]**	In vitro flow model and in vivo rabbit inferior vena cava thrombosis model	Endovascular low-frequency ultrasound combined with bifunctional microbubbles	Significantly improved clot dissolution and recanalization rates without endothelial damage.	Demonstrated potential of combined ultrasound-microbubble system for deep vein thrombosis (DVT) therapy.
15	**Chen J et al., *Front Bioeng Biotechnol*, 2023 [12]**	Rabbit inferior vena cava thrombosis model	Targeted microbubbles combined with low-power focused ultrasound	Achieved near-complete thrombus resolution	Validated targeted, low-intensity ultrasound-guided microbubbles as a safe and effective DVT treatment modality.

**Table 2:** Summary of Clinical Studies on Humans on Ultrasound-Mediated Thrombolysis.

No.	Study/Reference	Clinical Condition/Setting	Modality & Mechanism	Key Findings	Translational Relevance
1	**Alexandrov AV et al., *N Engl J Med*, 2004 [9]**	126 Acute Ischemic Stroke (AIS) patients (MCA occlusion)	Transcranial Doppler (TCD) Ultrasonography (2-MHz, continuous) + IV t-PA	TCD significantly augmented t-PA-induced arterial recanalization (49% vs. 30% for placebo; *p* = 0.03)	First major randomized trial (CLOTBUST) to demonstrate that non-invasive ultrasound can safely boost systemic thrombolysis for stroke.
2	**Slikkerveer J et al., *Ultrasound Med Biol*, 2012 [30]**	10 Acute ST-Elevation Myocardial Infarction (STEMI) patients/Prehospital	Sonothrombolysis (Pulsatile Ultrasound + Microbubbles + Alteplase)	No significant difference between treatment and control group in safety (minor adverse events 2/5 vs. 2/5, *p* = NS) and outcome (TIMI III flow 3/5 vs. 1/5 respectively, *p* = 0.23) was recorded	Pilot study to demonstrate study protocol is feasible and safe in acute cardiac setting
3	**Al-Terki H et al., *J Clin Med*, 2023 [13]**	20 Intermediate-High-Risk Pulmonary Embolism (PE) patients	Ultrasound-Accelerated Catheter-Directed Thrombolysis (USAT)	USAT improved echocardiographic measures of left ventricular function (e.g., RV/LV ratio decreased) and pulmonary arterial obstruction scores, with a relatively low complication rate.	Supports the safety and efficacy of local, low-dose thrombolysis combined with ultrasound to quickly relieve left heart strain in high-risk PE.
4	**Sterling KM et al., *Circ Cardiovasc Interv*, 2024 [31]**	489 Intermediate-High & High-Risk PE patients/Prospective Intl. Registry (KNOCOUT PE)	Ultrasound-Facilitated, Catheter-Directed Thrombolysis (US-CDT)	Significant reduction in RV/LV ratio by 0.49 at 24 h; 98.4% survival to discharge; low rate of major bleeding (1.7%).	Provides prospective, real-world evidence confirming the high efficacy and safety of US-CDT in a broad population of patients with severe PE.
5	**Prasad R et al., *J Vasc Access*, 2024 [32]**	Thrombosed Native Arteriovenous Fistula (AVF) (Dialysis Access)	Direct Percutaneous Thrombolyhsis (DPT) with Ultrasound Guidance + Urokinase	High technical success rate for salvaging thrombosed AVFs (84.2% for no-stenosis group, 97.5% for stenosis group followed by angioplasty).	Introduces DPT as a safe, economical, and minimally invasive technique for salvaging vital dialysis access sites

### 3.2. Microrobots in Thrombolysis

Microrobotic thrombolysis is a cutting-edge technique that uses tiny, untethered robotic devices to locate and dismantle blood clots through mechanical and chemical means (Table 3). These “microbots,” which range in size from micrometers to sub-millimeters, are directed to the site of a thrombus using external forces such as magnetic, acoustic, or optical fields. Their primary benefit lies in their ability to navigate the intricate vascular system, reaching and treating clots in narrow or highly curved vessels that are inaccessible to traditional catheter-based tools. In vivo studies in rats and rabbits have demonstrated that tPA-nanorobots can achieve near-complete recanalization of occluded veins within 40 min, restoring blood flow to nearly 100% of baseline levels [34].

A key milestone was achieved in 2024 by Pontius et al. who demonstrated magnetically driven “microwheels” composed of colloidal particles coated with tissue plasminogen activator (tPA) in a zebrafish model of arterial thrombosis [35]. Under a rotating magnetic field, these wheels assembled at the clot site, penetrated the thrombus, and released tPA locally—achieving effective recanalization with minimal haemorrhagic risk. Another breakthrough in 2023 came from Yang et al., who created swarming nanorobots coated with heparin-mimetic polymers and tPA [36]. These 300 nm particles self-assembled into motile femoral vein thrombosis model chains under magnetic fields, crawled to thrombi, and drilled into clots. In a rat, they achieved significant clot dissolution and restored blood flow, with no systemic toxicity or embolization. Several other innovative microrobotic strategies for thrombolysis are currently under investigation. One example includes ultrasound-driven microbots and chemically propelled nanomotors designed to break down clots [37]. At present, all of these technologies are still in the preclinical phase, having only been tested in laboratory settings or animal models. Human trials have yet to begin due to several major hurdles—such as ensuring the safety of the devices, maintaining precise control within the body’s dynamic vascular environment, enabling real-time imaging to monitor their movement, and navigating regulatory requirements for introducing such advanced tools into clinical practice.

**Table 3:** Key Preclinical and Translational Studies on Microrobots.

No.	Study/Reference	Model/Setting	Intervention & Mechanism	Key Findings	Translational Relevance
1	**Yang et al., *Sci Adv*, 2023 [36]**	In vivo rat femoral vein thrombosis	Swarming magnetic nanorobots coated with heparinoid-polymer brushes enabling anticoagulant surface and magnetic propulsion	Complete recanalization within 40 min; no haemorrhage or organ toxicity; effective under physiological flow	Demonstrated *biocompatible*, non-immunogenic magnetic nanorobot design suitable for translation; validates safety of swarm actuation in mammals
2	**Zhang et al., *Nat Commun*, 2023 [37]**	In vivo mouse tail thrombosis model and rat cerebral ischaemia/reperfusion injury model	Self-fuelled nano-penetrators composed of polyoxometalate–carbon composites that generate propulsion with endogenous H_2_O_2_	Achieved significant recanalization and improved neurological recovery without haemorrhagic complications.	First-in-class non-pharmaceutical, chemically self-driven electro-nanomechanical thrombolytic platform. Represents a paradigm shift from external energy or drug-dependent thrombolysis toward autonomous nanomechanical therapy; positions technology at TRL 4–5 (preclinical efficacy validated)
3	**Wang B et al., *Sci Adv*, 2024 [34]**	In vivo occlusion (rabbit carotid artery + rat femoral vein models)	tPA-anchored magnetic nanorobots (~300 nm Fe_3_O_4_ cores) propelled by rotating magnetic fields for localized fibrinolysis	Average recanalization time was 37 min, perfusion rates increased to ~100% after targeted therapy	First demonstration of autonomous magnetic nanorobots performing mechanical + enzymatic clot lysis in vivo; establishes scale-down feasibility for end-arterial applications
4	**Pontius et al., *PNAS*, 2024 [35]**	In vivo zebrafish thrombosis model	Magnetically powered “microwheels” composed of tPA-conjugated 4 µm magnetic particles rotating under external field	Microwheels recanalized occlusive thrombi within 30 min; tPA retention higher than diffusion-only controls	Provides real-time visualization of microrobotic swarm behavior and confirms efficacy of localized mechanical–enzymatic synergy

### 3.3. Electrothrombectomy: Harnessing Electrical Energy for Clot Removal

Electrothrombectomy is an emerging technique that combines mechanical thrombectomy with electrical energy to disrupt thrombi (Table 4 and Table 5). One standout example is the “eTrieve” system developed by Magneto Thrombectomy Solutions [38]. This catheter-based device utilizes the natural electrical properties of thrombi—specifically their negative charge—by applying a positive voltage at the catheter tip. This creates a strong electrostatic attraction, effectively anchoring the clot to the catheter for safe extraction or aspiration. The system’s design allows it to grasp and remove clots in one piece, even from deep or hard-to-reach vessels, while minimizing trauma to vessel walls. This technique can reduce the need for aggressive mechanical scraping, which lowers the risk of endothelial injury and clot fragmentation.

Preclinical studies have confirmed the device’s ability to firmly capture a wide range of clot types, including both soft, recent thrombi and denser, older fibrin-rich clots. In the first-in-human feasibility study (2021–2022) involving 10 patients with intermediate-to-high-risk PE, the eTrieve device was used under light sedation [39]. Results demonstrated successful clot removal in all cases, no device-related complications, and a 36% average reduction in the right ventricular/left ventricular diameter ratio within 48 h—reflecting significant hemodynamic improvement. The device successfully removed both large central and smaller peripheral emboli, offering complete revascularization. The combination of vacuum-assisted aspiration and electro adhesion appears to deliver thorough and efficient clot removal. However, the current evidence is limited to a small cohort, and larger, controlled studies are needed to validate its safety and efficacy. Device size also presents a limitation, as the original 20 F catheter is only suitable for large vessels. Future versions aim to be miniaturized for broader applications, such as treating strokes.

**Table 4:** Representative Preclinical Study on Electrothrombectomy.

No.	Study/Reference	Model/Setting	Intervention & Mechanism	Key Findings	Translational Relevance
1	**Magneto Thrombectomy Solutions,** ***Endovascular Today*, Biomed Israel Conference Press Release, 2022 [38]**	Preclinical and feasibility testing (bench and ex vivo clot models)	eTrieve™ Electrothrombectomy System—catheter applying localized positive voltage to attract and adhere to negatively charged thrombi for mechanical extraction.	Demonstrated strong electrostatic capture of thrombi of varying composition, enabling intact clot retrieval with minimal vessel trauma.	Proof-of-concept validation of a novel electroadhesion-based thrombectomy mechanism, supporting transition to human feasibility studies.

**Table 5:** Representative Clinical Study on Electrothrombectomy in Humans.

No.	Study/Reference	Model/Setting	Intervention & Mechanism	Key Findings	Translational Relevance
1	**Andersen A., Musialek P., Araszkiewicz A. et al., *J Am Coll Cardiol Intv*, 2023 [39]**	First-in-human, 10 patients with intermediate-risk pulmonary embolism	Mechanical–electric hybrid thrombectomy (eTrieve™ system) combining electro-adhesion and mechanical aspiration	100% procedural success and clot clearance Significant reduction in RV/LV ratio and pulmonary arterial pressure No major bleeding or device-related adverse events	Demonstrated safety and feasibility of photo-electric/electro-mechanical energy–assisted thrombus extraction; supports ongoing clinical development of hybrid device-assisted thrombolysis

### 3.4. Photothrombectomy: Light-Based Approaches to Clot Dissolution

Photothrombectomy utilizes light energy to induce thrombus formation and subsequent lysis (Table 6). In animal models, this technique has been explored as a method to selectively target and treat thrombi. By administering photosensitive agents that accumulate in thrombi, and then exposing the area to specific wavelengths of light, researchers can induce localized clot formation followed by targeted lysis. This approach allows for precise control over the treatment area, potentially reducing the risk of damage to surrounding healthy tissue. A widely used method is Excimer Laser Coronary Angioplasty (ELCA), which uses ultraviolet (308 nm) laser pulses to photoablatively vaporize clots and plaque in coronary arteries [40]. Studies have shown ELCA improves microvascular perfusion and reduces complications like no-reflow, especially in patients presenting late with ST-elevation myocardial infarction (STEMI). A recent trial of 319 STEMI patients found ELCA with PCI significantly improved myocardial blush grades and reduced distal embolization [41].

Beyond coronary use, ELCA has also been applied to peripheral artery and venous thrombi, where it effectively clears clots when used with aspiration or medication. Though laser devices are generally safe, caution is required to avoid vessel damage by adhering to operational guidelines. In parallel, researchers are developing photo-activated nanomaterials for less invasive photothrombectomy. These include nanoparticles that absorb near-infrared (NIR) light and convert it into localized heat (photothermal therapy), softening or fragmenting clots. A 2023 study showcased a NIR-II responsive nanoparticle carrying a nitric oxide (NO) donor [42]. In animal models, laser exposure triggered heat and NO release, resulting in near-complete clot dissolution without systemic drugs. These light-based methods are still in preclinical stages but offer unique advantages—localized, on-demand clot lysis with minimal systemic exposure, reduced bleeding risks, and potential for theranostics (simultaneous imaging and therapy). Challenges include deep tissue light delivery and ensuring nanoparticle specificity to avoid off-target effects.

**Table 6:** Representative Preclinical and Early Clinical Studies on Photothrombectomy.

No.	Study/Reference	Model/Setting	Modality & Mechanism	Key Findings	Translational Relevance
1	**Jawad-Ul-Qamar M. et al. *Open Heart*, 2021 [40]**	50 patients undergoing elective or emergency PCI	Excimer laser coronary angioplasty (ELCA) using 308 nm ultraviolet laser pulses to photo-ablate fibrin and platelet aggregates	- High procedural success with acceptable complication rate - Reduced residual thrombus burden; improved distal flow	Demonstrated clinical feasibility of photochemical–photothermal ablation of thrombus within coronary circulation
2	**Kujiraoka et al., Yoshida K., Fukamizu S. *Lasers Med Sci*, 2023 [41]**	Clinical series, 319 patients with STEMI	ELCA-assisted primary PCI for thrombotic occlusions	- Shorter procedural times and improved TIMI flow when ELCA used in early presentation (<3 h)—Comparable safety vs. standard PCI	Confirms ELCA’s role in acute coronary thrombosis; provides translational link from photothermal ablation to modern endovascular reperfusion strategies
3	**Song J et al., *Nat Commun*, 2023 [42]**	In vitro & in vivo mouse carotid artery thrombus model	Fibrin-specific homopolymer nanoparticles with NIR-II photoacoustic imaging + photo-triggered thermal release	Precise NIR-II-induced clot disintegration; dual imaging and therapy with minimal tissue heating	Demonstrates fully integrated imaging–therapy (PA/photothermal) system for real-time thrombus monitoring

### 3.5. Magnetic Targeted Thrombolysis: Iron Based Intravascular Therapy

Iron based systems have been demonstrated to be a potentially feasible emerging thrombolytic technology (Table 7). These iron oxide (IO) containing nanoparticles can serve as carrier of thrombolytic agent and have been employed for their magnetic properties, enabling precise delivery of thrombolytic agents to the site of thrombosis [43]. As a result, these technologies may reduce systemic exposure to thrombolytic agent and thus reduce the bleeding risk. They may also enhance clot lysis efficiency.

Various iron oxide (IO) containing technologies have been demonstrated in in-vitro and animal models to be an effective thrombolytic agent. Some examples include silver-iron oxide nanoparticles (AgIONPs) [44], complexes containing erythrocyte-membrane camouflaged nanoparticles with incorporation of ultra-small IO and urokinase [43], liposomal nanovesicle containing IO-perfluorohexane-urokinase [45], nanoparticles containing glycol chitosan, polypyrrole, IO and heparin [46], discoidal polymeric particles loaded with recombinant tissue plasminogen activator (rtPA) and IO nanoparticle [47], IO nanoparticles combined with an antibody recognizing activated integrin αIIbβ3 [48], rTPA modified magnetite nanoparticles [49], and plasma-derived theranostic platelet vesicle incorporating iron oxide constructed nano-propellers [50]. IO-based composites have also been engineered for dual anticoagulant and thrombolytic functions [45]. This dual functionality approach can potentially improve therapeutic outcomes in dissolution and prevention of thrombus. These materials provide controlled release of thrombolytic drugs, ensuring sustained efficacy in dissolving clots. A different dual-functionality IO-containing nanoplatform that incorporates urokinase and has near-infrared mediated photothermy shows good thrombolytic potential [51]. Some of these IO containing composites can even exhibit simultaneous diagnostic and therapeutic properties [52], enabling precise imaging and delivery of thrombolytic agents to the site of thrombosis.

In addition, MRI-detectable IO-containing system has also been developed. Polydopamine-coated IO-containing nanoparticles have been incorporated into MRI-compatible thrombolytic systems, enabling real-time monitoring of thrombus dissolution and therefore potentially improving therapeutic efficiency [53]. Other polydopamine-based composites are capable of binding integrin αIIbβ3 and P-selectin on activated platelets concurrently to improve the targeting of the composites to thrombus [54]. This leads to enhancement in the imaging of thrombus in vivo. While these IO-based developments are exciting, in vivo data of the application of these technologies in humans is eagerly awaited.

**Table 7:** Representative Preclinical Studies on Magnetic Targeted Thrombolysis.

No.	Study/Reference	Model/Setting	Modality & Mechanism	Key Findings	Translational Relevance
**1**	**Zhang Y et al., *Int J Nanomedicine*, 2019 [54]**	In vitro simulated circulatory device & in vivo mixed thrombus mouse model	Polydopamine-modified dual-ligand NPs for MRI/PA dual-modality imaging	High fibrin affinity and strong imaging contrast	Early example of theranostic dual-imaging thrombus agents
**2**	**Zhang Y et al., *ACS Appl Mater Interfaces*, 2021 [51]**	In vitro clots & in vivo rat thrombosis model	MOF-derived carbon nanoplatforms with multimodal (photo + magnetic) capabilities	Enhanced fibrin breakdown with optical tracking; low bleeding risk	Early MOF-based multimodal template for current hybrid designs
**3**	**Choi W et al., *Biomater Res*, 2022 [47]**	In vivo photothrombotic stroke (mouse)	Magneto-acoustic particles targeted to occlusion site	Restored cerebral perfusion to ~80% baseline; improved neurological score; no haemorrhage	Extends magneto-acoustic synergy to neurovascular (stroke) models
**4**	**Tang X et al., *Small*, 2022 [49]**	In vivo rat venous thrombosis	Enzyme–magnetite nanoparticle swarms for low-dose pharmacomechanical thrombolysis	Tenfold thrombolytic efficiency achieved when compared to pure rTPA	Demonstrates enzyme-magnetic swarm synergy for dose minimization
**5**	**Cabrera D et al., *J Thromb Haemost*, 2022 [48]**	In vitro human plasma clots	Magnetic hyperthermia using clot-targeted Fe_3_O_4_ NPs	Local heating (≈42 °C) permeabilized fibrin, increasing tPA susceptibility	Establishes adjunctive magnetothermal pre-conditioning concept
**6**	**Liu KT et al., *Adv Healthc Mater*, 2023 [46]**	Microfluidic & in vivo rodent models	Self-indicating biomimetic nanoassembly with site-specific photothermal activation	Real-time optical signal correlates with local clot lysis; reduced systemic exposure	Adds theranostic self-reporting functionality for precision feedback
**7**	**Jheng PR et al., *Mater Today Bio*, 2023 [50]**	In vitro + in vivo murine thrombosis model	Cold-plasma–enabled platelet-vesicle iron-oxide nano-propellers	Active rotation and localized heating achieved near-complete clot removal; biocompatible	Introduces bio-hybrid nano-propeller concept leveraging plasma activation
**8**	**Vazquez-Prada KX et al., *Small*, 2023 [52]**	In vivo mouse thrombosis model	Spiky Ag–Fe_3_O_4_ nanoparticles for targeted photothermal therapy + multimodal imaging	Local temperature rise ≈ 45 °C induced thrombus ablation within 5 min; high imaging contrast	Improved photothermal efficiency and MRI/PA visibility for precision targeting
**9**	**Ruan R et al., *Adv Healthc Mater*, 2024 [43]**	In vitro 3D printed vein vasculature model & in vivo rat thrombosis model	Targeting nanomotor with NIR + ultrasound dual-triggered transformation for staged cascade thrombolysis	Achieved multistage propulsion and complete recanalization; enhanced safety by sequential energy activation	Introduces polystage cascade paradigm combining NIR and US energy cues
**10**	**Zhu L et al., *Adv Healthc Mater*, 2024 [45]**	In vivo mouse tail vein thrombosis model	Erythrocyte-membrane-camouflaged magnetic nanocapsules with photothermal + magnetothermal dual modes	Rapid thrombus clearance; prolonged circulation; no organ toxicity	Biomimetic stealth carrier combines immune-evasion + dual heating
**11**	**Jacqmarcq C et al., *Nat Commun*, 2024 [53]**	In vivo stroke mouse model	Polydopamine-coated iron-oxide nanoparticles enabling MRI detection of microthrombi	High-resolution MRI tracking of microthrombi; potential for targeted therapy	Provides diagnostic integration layer for image-guided nano-thrombolysis
**12**	**Vazquez-Prada KX et al., *Biomater Sci*, 2025 [44]**	In vitro human blood clot and thrombosis mouse model	Branched Ag–Fe_3_O_4_ nanoparticles enabling drug-free magnetothermal ablation	Localized hyperthermia disrupted fibrin mesh within minutes without lytic drug	First demonstration of purely physical, magnetothermal thrombolysis—bleeding-sparing concept

### 3.6. Nanoparticle Technology

**Figure 2:**
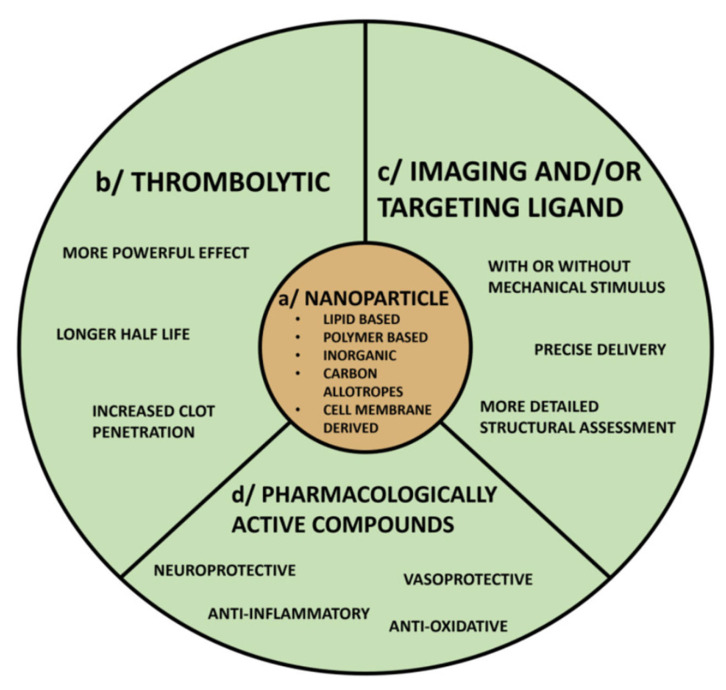
Conceptual design of a nanoparticle-based thrombolytic agent combining (**a**) a nanocarrier platform with (**b**) a thrombolytic payload, (**c**) targeting or imaging ligands, and (**d**) additional therapeutic compounds. Such multi-functional nanomedicines aim to prolong drug half-life, increase clot penetration, enable precise delivery (potentially with mechanical triggers), and concurrently address secondary injury.

Nanotechnology (Figure 2) offers a promising solution by concentrating thrombolytic activity at the clot site and minimizing exposure elsewhere [55] (Table 8). Encapsulation or conjugation of thrombolytic drugs onto nanoparticles (NPs) can prolong circulation half-life, protect the drug from degradation, and temporarily attenuate activity during circulation [56]. This allows more of the thrombolytic drug to reach the thrombus before activation, lowering the effective dose and reducing bleeding complications.

Nanocarriers can also be functionalized for targeted binding (e.g., fibrin- or platelet-specific ligands) or designed for stimuli-responsive release (e.g., ultrasound, magnetic fields). Additionally, multifunctional “theranostic” platforms (portmanteau of therapeutic and diagnostic used in context of nanomedicine is one that combines both treatment and imaging capabilities in a single agent or device) can co-deliver imaging tracers or adjunctive therapeutics to mitigate ischemia–reperfusion injury [57].

**Table 8:** Representative Preclinical and Early Clinical Studies on Nanoparticle technology.

No.	Study/Reference	Model/Setting	Modality & Mechanism	Key Findings	Translational Relevance
1	**Chung TW et al., *Biomaterials*, 2008 [58]**	In vitro blood clot model	Chitosan-coated plasminogen activators in PLGA nanoparticles	Sustained release of tPA; accelerated clot lysis with reduced bleeding risk	One of the earliest controlled-release nanocarrier approaches for thrombolysis
2	**Korin N et al., *Science*, 2012 [59]**	Microfluidic and in vivo mice model	Shear-activated nanotherapeutics that unfold under pathologic shear	Selective tPA release in occluded vessels → site-specific clot lysis without systemic bleeding	Pioneering “smart release” platform for occlusion-responsive thrombolysis
3	**Colasuonno M et al., *ACS Nano*, 2018 [60]**	Microfluidic chip and in vivo murine thrombosis model	Erythrocyte-inspired discoidal polymeric nanoconstructs carrying tPA	2× faster clot lysis vs. free tPA; prolonged circulation	Validated biomimetic nanoplatform with enhanced hemodynamic stability
4	**Blum NT et al., *ACS Appl Mater Interfaces*, 2019 [61]**	In vitro clot and HIFU setup	Phospholipid-coated hydrophobic mesoporous silica NPs to enhance HIFU thrombectomy	Improved lysis with low debris generation	Demonstrates safe energy–nanoparticle coupling
5	**Refaat A et al., *J Control Release*, 2021 [62]**	In vitro halo-clot model	NIR-responsive liposomes for protein delivery	Triggered on-demand release by light; no off-target effect	Prototype for controlled, non-systemic activation
6	**Hu L et al., *Int J Nanomedicine*, 2022 [63]**	In vitro and rat model	Hybrid nanoplatform combining mechanical ultrasound blasting and drug delivery	Synergistic mechanical + pharmacologic lysis with reduced dose	Bridges mechanical and nano-drug approaches
7	**Yu W et al., *Acta Biomater*, 2022 [64]**	Mouse ischemic stroke model	Biomimetic nanovesicles mimicking platelet membrane for thrombus targeting + ischemia-reperfusion protection	Enhanced clot lysis and post-ischemic tissue repair	Dual-action (blood–brain barrier and reperfusion protection) platform
8	**Chen YT et al., *ACS Appl Mater Interfaces* 2023 [65]**	In vitro + in vivo rodent thrombosis model	Biomimetic platelet nanomotors for site-specific thrombolysis	Autonomous motion toward thrombus; reduced reperfusion injury	Proof of concept for biohybrid motile nanoplatforms
9	**Wang Z et al., *J Nanobiotechnol*, 2024 [66]**	In vivo rat arterial thrombosis	Dual-mode nanoprobe integrating ultrasound + NIR activation	Synergistic lysis with enhanced targeting and monitoring	Validates multi-energy dual-mode activation strategy
10	**Yin L et al., *Nat Mater*, 2024 [67]**	In vivo mouse arterial and venous thrombosis models	Intelligent DNA nanodevice for precision thrombolysis	The device actively binds and penetrates the thrombus; achieves complete clot removal in vivo with low bleeding risk due to enzyme-free action.	Major advance in active, non-enzymatic thrombolysis using programmable DNA nanotechnology

### 3.7. Types of Nanomaterials for Thrombolysis and Mechanism of Action

**Figure 3:**
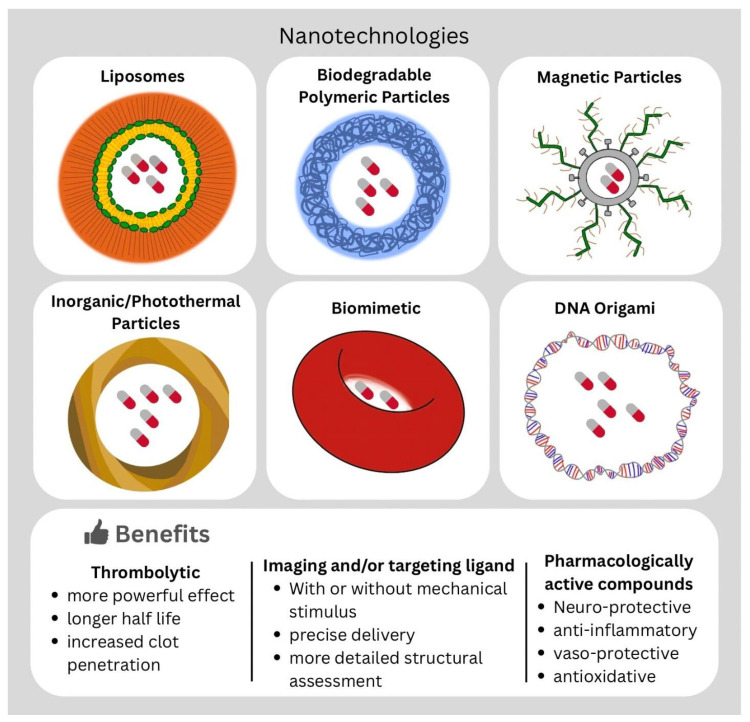
Existing nanotechnologies and potential translational benefits for thrombolysis. DNA (deoxyribonucleic acid).

Various nanocarrier platforms have been investigated for targeted thrombolytic delivery (Figure 3):

1. Lipid-Based Nanocarriers:

Polyethylene glycol (PEG)ylated liposomes (~145 nm) encapsulating tPA significantly extend circulation half-life—up to ~132 min versus ~6 min for free tPA—while preserving enzymatic activity and accelerating in vivo clot lysis by approximately 50% [68]. Surface grafting of fibrin-targeting peptides such as CREKA further enhances thrombus localization: in one study CREKA-functionalized nanoparticles showed ~3.1-fold greater thrombus binding in vitro compared to non-targeted controls [63].

2. Biodegradable Polymeric Nanoparticles:

Poly(lactic-co-glycolic acid) (PLGA) and chitosan-based NPs (~200 nm) provide controlled release and protect fibrinolytics from degradation. Chitosan-coated PLGA NPs maintain ~80% tPA activity after 24 h and achieve 40% faster in vitro lysis [58]. Shear-activated aggregates disassemble under pathological shear stress, restoring flow at reduced tPA doses [59].

3. Magnetic Nanoparticles:

Polyacrylic acid-coated magnetic nanoparticles (PAA-MNP) conjugated to recombinant tPA are enabled by magnetic guidance, restoring ~82% of baseline flow in rat models while requiring <20% of the standard tPA dose [69]. Dynamic magnetic actuation (rotating fields) mechanically disrupts fibrin, augmenting biochemical thrombolysis.

4. Inorganic and Photothermal Nanoparticles:

Inorganic and photothermal nanoparticles have shown potential in thrombolytic therapy by enhancing precision through spatially controlled and efficient clot resolution. These systems exploit materials like gold nanoshells and mesoporous silica, which generate localized heat under NIR light. NIR-responsive liposomes, for example, can release therapeutic proteins on demand, effectively dissolving clots while limiting systemic fibrinolysis and reducing hemorrhagic risk [62]. Phospholipid-coated, hydrophobically modified mesoporous silica nanoparticles have demonstrated enhanced clot disruption when used in conjunction with HIFU, capitalizing on both thermal and mechanical mechanisms to augment thrombolysis [61]. More recently, dual-mode nanoprobes combining ultrasound and NIR light have enabled targeted, synergistic thrombolysis, improving recanalization while minimizing systemic side effects [66].

5. Biomimetic Nanocarriers:

Biomimetic nanocarriers cloaked in platelet or erythrocyte membranes harness natural thrombus homing and immune-evasion capabilities to improve thrombolytic delivery. In one study, platelet membrane-coated vesicles encapsulating tPA and melanin (tPA/MNP@PM) used platelet-driven targeting and NIR-triggered release to accelerate thrombolysis and reduce reperfusion injury in AIS models [64]. Another innovative design utilized platelet-mimetic nanomotors that combine platelet membrane coatings with active propulsion: these constructs achieved precise, site-specific clot dissolution and ischemic lesion protection [65]. Discoidal erythrocyte-inspired constructs double circulation time and enhance lysis [60].

6. DNA Origami Nanodevices:

DNA origami assembles long single-stranded scaffolds into precise 3D shapes. A recent study by Yin et al. introduced a thrombin-responsive DNA origami nanodevice designed from tubular DNA origami structures that encapsulate tPA and releases it only when thrombin levels exceed a pathological threshold. In animal models of stroke and pulmonary embolism, tPA–DNA nanodevices demonstrated superior thrombolytic efficacy, prolonged circulation time, and reduced bleeding compared to free tPA [67].

## 4. Discussion: Advancing Thrombolysis Through Precision Platforms

The landscape of thrombolytic therapy is being reshaped by six distinct technological platforms, each offering unique mechanisms, advantages, and clinical maturation pathways (Table 9). Ultrasound-mediated thrombolysis (UMT) stands as the most clinically advanced technology in thrombectomy, leveraging acoustic cavitation to potentiate clot dissolution. The efficacy of UMT lies in its dual physical mechanisms: stable cavitation creates microstreaming forces that permeabilize thrombi for drug penetration, while inertial cavitation generates shockwaves that cause mechanical fragmentation [17]. Clinically, catheter-based UMT systems like EKOS have received United States Food and Drug Administration (FDA) clearance for pulmonary embolism and deep vein thrombosis, as evidenced by its use across 64 international sites in the recent KNOCOUT PE registry. This reflects significant global experience, with the system demonstrating superior clot resolution and reduced treatment duration compared to anticoagulation alone [31]. However, transcranial applications remain limited partly due to skull attenuation in 15–20% of patients, which necessitates personalized calibration of acoustic parameters therefore limiting widespread adoption. Several sonothrombolysis trials [8,9,29] have also produced variable results due to inconsistent ultrasound delivery parameters—particularly acoustic intensity, duty cycle, and insonation geometry—which directly affect cavitation dynamics and thrombus permeability. Operator-dependent variability and differences in skull bone thickness or vascular anatomy further contributed to heterogeneous recanalization rates. These limitations underscore the need for standardized acoustic protocols and real-time feedback control to ensure reproducible outcomes across patient populations. Although the CLOTBUST trial demonstrated that combining intravenous tPA with transcranial Doppler significantly improved early recanalization in AIS, the larger and more recent CLOTBUST-ER trial which used an automated headframe to deliver ultrasound did not show improvement in 90-day functional outcomes [70]. The future clinical utility of transcranial sonothrombolysis is likely contingent on the development of patient-specific dosing protocols and improved targeting, potentially guided by real-time imaging.

In contrast, electrothrombectomy represents an emerging mechanical advancement over traditional thrombectomy. Devices like the eTrieve catheter exploit the natural electronegativity of thrombi, using positive-voltage tips to adhere to and extract intact clots, minimizing fragmentation and distal embolization [38]. Early human trials in intermediate-high-risk pulmonary embolism have demonstrated 100% technical success with rapid hemodynamic improvement, such as a 36% reduction in the RV/LV ratio within 48 h [39]. While the current 20F profile restricts its use to large vessels, miniaturization efforts are underway to enable neurovascular applications. The clinical readiness of electrothrombectomy is intermediate: pilot studies confirm feasibility, but randomized trials against standard mechanical thrombectomy are still required to establish definitive clinical outcomes.

On the innovative frontier, microrobotic systems remain preclinical but hold substantial promise. These submillimeter devices, including magnetically driven “microwheels” and heparin-mimetic nanorobots, navigate tortuous vasculature inaccessible to conventional catheters. Under external magnetic fields, they penetrate clots mechanically and release thrombolytics locally, restoring over 90% blood flow in rodent models within 40 min [34]. Despite their micro-scale precision, these systems face challenges related to biocompatibility (such as metallic nanoparticle retention), real-time tracking limitations, and manufacturing scalability, which delay their transition to human trials. Magnetic targeted thrombolysis leverages iron oxide nanoparticles as drug carriers, guided by external magnets. In preclinical studies, platforms like fibrin-targeted IO-rtPA conjugates have reduced tPA doses by 80%, enabling MRI-based therapy monitoring. However, unresolved concerns regarding immunogenicity prevent initiation of human trials. Similarly, photothrombectomy, which combines energy-based and nanotechnological approaches, shows promise. (ELCA) is already established in coronary interventions, where it photo ablates clots using 308 nm ultraviolet light, reducing no-reflow phenomena in late-presenting STEMI patients [41]. Meanwhile, emerging near-infrared(NIR)-responsive nanoparticles, such as NO-releasing or photothermal agents, aim to dissolve clots without the use of systemic drugs [42]. These nanoplatforms have demonstrated exceptional spatial control in animal models, but they face light-penetration challenges in deep tissues. The clinical adoption of ELCA is well-established in coronary work, while light-activated nanoparticles are 3–5 years from first-in-human trials.

Finally, multifunctional nanocarriers showcase biochemical ingenuity. Biomimetic designs, such as platelet membrane-coated vesicles and erythrocyte-inspired discoidal particles, enhance thrombus targeting and circulation half-life. DNA origami nanodevices offer precision-controlled thrombolysis by releasing tPA only at clot sites where thrombin levels are pathologically elevated [67]. While all these platforms remain preclinical, they offer unique approaches to addressing reperfusion injury via the co-delivery of cytoprotective agents. Among these, lipid-based carriers like CREKA-targeted liposomes lead in translational progress, with Phase I trials anticipated by 2026.

Collectively, these emerging thrombolytic technologies have the potential to influence future guidelines on clot dissolution and revascularization. By improving lytic precision and reducing haemorrhagic risk, modalities such as ultrasound-assisted, microrobotic, magnetic, and nanoparticle-based systems could shape the evolution of precision and device-assisted thrombolysis frameworks. As these innovations progress from preclinical validation to early clinical translation, standardized evaluation and regulatory integration will be essential to guide their adoption into next-generation therapeutic algorithms.

**Table 9:** Comparison of six emerging thrombolysis technologies across efficacy, clinical readiness, benefits, risks, and representative systems.

Technology	Core Mechanism	Evidence/Efficacy Signal	Technology Readiness Level *	Key Benefits	Potential Clinical Risks	Translational Barriers	Representative Platforms
**Ultrasound-Mediated Thrombolysis (UMT)**	Acoustic cavitation (stable + inertial) enhances drug penetration & mechanical clot disruption; catheter or external US.	↑ Recanalization in stroke (CLOTBUST); RV strain reduction & clot debulking in PE/DVT (EKOS); lower lytic dose requirements.	TRL 9	Targeted thrombolysis with reduced systemic tPA; adaptable (neuro, venous, coronary); can shorten ICU time.	Vessel injury if high energy; embolic debris; haemorrhage if parameters mis-set; skull attenuation limits transcranial use.	Parameter standardization; access cost; operator training; patient-specific acoustic planning (bone, body habitus).	EKOS™; CLOTBUST protocols; histotripsy/microtripsy variants (investigational).
**Electrothrombectomy**	Positively charged catheter tip electro-adheres to negatively charged thrombus for intact extraction ± aspiration.	Small PE feasibility series: 100% technical success; ~36% RV/LV reduction at 48 h; broad clot phenotype capture.	TRL 3–4	Intact clot removal; minimal fragmentation; drug-sparing (reduced bleeding); rapid hemodynamic response.	Large-bore access (20F) bleeding; vascular trauma at access; incomplete capture in branching anatomy; electrical malfunction (rare).	Miniaturization for smaller vessels; randomized outcome trials; comparative cost-effectiveness; electrical safety standards.	eTrieve™ electro-adhesion catheter
**Microrobots**	Untethered magnetic/acoustic micro- or nanorobots that navigate small, tortuous vessels; mechanical boring + local drug release.	Rodent/zebrafish models: near-complete flow restoration within ~40 min; effective tPA delivery at low dose.	TRL 6	Access to distal microvasculature; ultra-localized therapy; potential multiplex (drug, sensors).	Off-target lodging; immune/toxic material response; uncontrolled migration; retrieval failure; microembolization.	Real-time tracking & control in humans; biocompatible, biodegradable materials; scalable manufacture; novel regulatory pathway.	Magnetically driven “microwheels”; heparin-mimetic swarming nanorobots; ultrasound-driven nanomotors (experimental).
**Photothrombectomy**	Light energy (UV laser photoablation; NIR photothermal/photochemical activation of clot-bound agents) to vaporize or soften thrombus.	ELCA improves myocardial blush/reduces no-reflow in late STEMI; peripheral thrombus debulking reports; NIR nanoparticles dissolve clots in animals without systemic lytics.	TRL 4 (NIR nanoparticles); TRL 9 (ELCA)	Precise on-demand lysis; low systemic drug use; adjunct to PCI; theranostic potential with photoactive probes.	Thermal or photochemical vessel injury; perforation if mis-fired; need for line-of-sight/fiber delivery; off-target activation of photosensitizers.	Efficient deep-tissue light delivery; selective targeting; device cost/training; regulatory clearance for new photo-agents.	ELCA (excimer laser, coronary); NIR-responsive NO or photothermal nanoparticles (preclinical).
**Magnetic Targeted Thrombolysis**	Superparamagnetic iron-oxide (SPIO) particles carrying lytics steered & concentrated at clot via external magnets; can add mechanical oscillation.	Animal data: effective lysis with ≤20% standard tPA dose; ~80% dose reduction shown in models; MRI-trackable constructs.	TRL 4	Major lytic dose reduction (lower bleeding); image-guided targeting; combinable with mechanical or thermal triggers.	Particle aggregation/microvascular obstruction; complement activation; iron overload or RES sequestration; magnet mis-targeting.	Human-scale magnetic steering hardware; GMP nanoparticle production; long-term biodistribution safety; combo product regulation.	Fibrin-targeted IO-rtPA conjugates; erythrocyte-camouflaged magnetic nanocapsules; magnetoacoustic particles.
**Nanotechnology (Targeted/Stimuli-Responsive Nanocarriers)**	Drug (tPA/urokinase) loaded in liposomes, polymers, biomimetic or DNA origami constructs; clot-targeting ligands; triggered release (shear, US, pH, NIR, enzymes).	Liposomal tPA: ~22× half-life extension; ~50% faster lysis; CREKA targeting ~3× clot uptake; polymer NPs preserve 80% activity/↑ lysis; DNA origami reduces hemorrhage ~70% in stroke models.	TRL 4–5	Lower systemic exposure/bleeding; prolonged circulation; multi-cargo (cytoprotectants, imaging); customizable to clot biology (arterial vs. venous).	Material toxicity; immune recognition; off-target deposition (RES, lung); variable clot penetration; manufacturing heterogeneity.	GMP scale-up; regulatory path as drug–device combo; stratified clinical trial design; cost of complex biologics; personalized targeting biomarkers.	CREKA-tPA liposomes; platelet-membrane nanovesicles; shear-activated nano-aggregates; thrombin-responsive DNA nanodevice.

* Technology Readiness Levels (TRLs) provide a standardized framework for assessing the maturity of biomedical innovations. TRL 1 represents basic scientific research, where fundamental principles are observed without specific application. TRL 2 involves the formulation of technology concepts and hypotheses. At TRL 3, proof-of-concept is established through experimental models, typically in vitro. TRL 4 denotes validation in small-animal models, while TRL 5 involves translational studies in large animals and initial Good Manufacturing Practice (GMP) preparations. First-in-human trials begin at TRL 6, assessing feasibility and preliminary safety. TRL 7 corresponds to pivotal, often multicenter clinical trials that establish therapeutic efficacy. Technologies that have achieved regulatory approval (e.g., FDA, EMA) are designated TRL 8. Finally, TRL 9 indicates full clinical integration, with widespread use in healthcare settings and ongoing post-marketing surveillance. Adapted from: U.S. Department of Health and Human Services, NIH Office of Science Policy (2017); European Commission Horizon 2020 TRL Definitions. *DNA* deoxyribonucleic acid, *DVT* deep vein thrombosis, *ELCA* Excimer Laser Coronary Angioplasty, *EKOS* EkoSonic Endovascular System, *ICU* intensive care unit, *IO-rtPA* iron oxide-recombinant tissue plasminogen activator, *LV* left ventricle, *NIR* near-infrared, *NO* nitric oxide, *PCI* percutaneous coronary intervention, *PE* pulmonary embolism, *RES* reticuloendothelial system, *RV* right ventricle, *STEMI* ST elevation myocardial infarction, *tPA* tissue plasminogen activator, *UV* ultraviolet, ↑ increased.

The path forward demands synergistic integration of these platforms. Hybrid approaches—such as combining UMT with magnetic nanoparticle guidance—could overcome tissue-specific barriers like skull attenuation while enhancing clot penetration. Concurrently, the field must prioritize real-time theranostic feedback, embedding imaging tracers within nanocarriers to enable PET-MRI monitoring of lysis efficacy and reperfusion injury, thus allowing dynamic treatment adjustment. Personalization will be equally critical: artificial intelligence algorithms analyzing thrombus composition (CT/MRI-derived fibrin/platelet ratios) could guide modality selection—for instance, deploying mechanical strategies for fibrin-rich clots versus photothrombectomy for platelet-dominant thrombi. The integration of artificial intelligence (AI) for real-time analysis of clot lysis and treatment efficacy is an emerging frontier, promising to enable dynamic adjustment of thrombolytic strategies for true personalized therapy. Regulatory innovation must parallel technical advances, with accelerated pathways for “hybrid devices” (e.g., nanocarriers + ultrasound activators) under existing frameworks like the FDA’s Breakthrough Device designation. Emerging evidence suggests that baseline ADAMTS13 and von Willebrand factor (vWF) activity may influence clot composition and lysis responsiveness. Integrating these biomarkers with these novel thrombolytic technologies may lead to better patient stratification and prediction of therapeutic outcomes. Crucially, clinical trials must evolve beyond recanalization metrics to capture long-term functional outcomes—neurologic recovery in stroke, right ventricular function in PE, and amputation-free survival in peripheral artery disease. Only through such comprehensive validation can these technologies transition from promising tools to transformative standards of care. Finally, future research should aim to clarify which patient and thrombus characteristics best predict benefit from device-enabled thrombolysis, determine the minimal effective thrombolytic dose or energy threshold to balance efficacy and bleeding risk, and compare long-term functional recovery across different vascular territories. To achieve this, upcoming clinical trials should be multicenter, randomized, and outcome-driven, incorporating standardized imaging, biomarker stratification, and long-term endpoints such as survival, organ function, and quality of life. Adaptive platform trial designs are encouraged to allow parallel evaluation of emerging technologies within unified frameworks.

## 5. Conclusions

The global burden of thrombotic diseases demands a paradigm shift toward precision-targeted, minimally invasive thrombolysis. Innovations like ultrasound-mediated lysis, microrobots, electrothrombectomy, and multifunctional nanocarriers leverage mechanical, acoustic, electrical, and photonic energy—often combined with bioengineered targeting—to dissolve clots with unprecedented specificity while reducing bleeding risks. These advances, including catheter-based systems (e.g., EKOS) and theranostic platforms, promise to redefine clinical guidelines across stroke, PE, and beyond. However, clinical translation requires overcoming hurdles in device miniaturization, large-scale validation, and emergency workflow integration. Interdisciplinary collaboration is essential to ensure these technologies not only restore perfusion but also mitigate reperfusion injury, ultimately ushering in an era of personalized thrombolysis that reduces global morbidity and mortality.

## Data Availability

Data sharing not applicable to this article as no datasets were generated or analysed during the current study.
